# News and Events of the Week

**Published:** 1910-02-26

**Authors:** 


### News and Events of the Week.

Mr. James Fenn Clark, M.R.C.S., of Beauchamp Square, Leamington, Warwick, who died in January at the age of 87, left estate valued at £22,701 gross, and £21,859 net.

The Tuberculosis Exhibition organised by the National Association for the Prevention of Consumption was opened at the Alexander Institute, Carter Street, Walworth Road, S.E., on Thursday, February 17, at 5 p.m., the Rev. Canon Horsley, M.A., the Mayor of Southwark, in the chair.

The vital statistics of the Hampstead Garden Suburb for the year are : death-rate, 1.6 per 1,000 (three out of the four persons who have died being over 65 years of age); birth-rate, 13.5 per 1,000; and for infant mortality rate *nil* —not one of the 33 babies born on the estate having died. When we remember that the death-rate is 14.5 for all London, and the infant mortality in certain districts is 180 per 1,000, the Garden Suburb figures are eloquent indeed.

An interesting letter by Mr. O. Waddington appears in a recent issue of *The Chemist and Druggist* on the chemist and druggist, from which we take the liberty of quoting the following excerpts, as they are of interest to medical men as well as to pharmacists : " It is the tragic writer who has paid most attention to the members of our craft. True, it is almost always cupboard love that he has shown us—if there is an apothecary introduced into a work of fiction you may be sure that the author has an eye on his poison cupboard, and sooner or later it will mean more work for the coroner. But these chemists of romance usually exhibit small concern for the coroner. There is a typical instance in Stephen Phillips's ' Paolo and Francesca,' where the old apothecary says :

> ' I will not sell to murder,
> But unto any weary of their life
> I sell a painless issue out of it.'

Scarcely a workable principle nowadays! This poison-selling specialist is more or less of a stock figure in romance. He is usually depicted as a man well on in years and addicted to miserliness. The ranks of pharmacy are not lacking in pessimists nowadays, but they have not seen nor imagined anything so wretched as the apothecary in ' Romeo and Juliet.'

The work of men such as Henrik Ibsen and H. G. Wells, whose early life was devoted to pharmacy, provides an engrossing subject for study with a view to estimating the effect of these early experiences upon their writings. Dr. Rank in ' A Doll's House ' and Oswald Alving in ' Ghosts ' might well be artistically moulded memories of Ibsen's early days as an apothecary's apprentice at Grimstad, of which some interesting details are given in the biography by H. Jaegar. There is another class of authors which might also claim our special interest; those who have been addicted to drug habits. The pre-eminent example of this class is, of course, De Quincey, whose ' Confessions of an English Opium Eater ' should be on the bookshelf of every literary chemist. It is the pharmacist's lot to spend long days in his shop. Is it not worth while for him to read such things as Robert Browning's poem, ' The Laboratory,' and thereby have his eyes opened to the fact that his surroundings are not so drab as he previously thought? Is it not worth while for him to cultivate an imaginative insight into the lives of his fellowmen? Surely imagination and insight are not to be despised, even as business assets."

Dr. J. Bullock, who has been resident medical officer of the Prince Alfred Hospital, Sydney, has been elected a Rhodes scholar and entered as a student at Oxford.

Professor Arthur Keith, M.D., devoted the second of his series of Hunterian lectures on " The Anatomy and Relationship of the Negro and Negroid Races," at the Royal College of Surgeons, to the evidence of the existence of a negro race in Europe in ancient times. There was, he said, no longer any doubt as to the truth of the conclusion arrived at by Dr. Verneau that the southern part of Europe, at least, was occupied by a negro race about 60,000 years ago.

The next course of post-graduate lectures under the auspices of the Berlin Dozenten Vereinigung commences on February 28. It is a monthly course, and a very interesting syllabus has been issued. Only qualified practitioners are allowed to subscribe to the course, the fee for each set of lectures and practical work ranging from 20 marks (£1) to 100 marks (£5). We can thoroughly recommend these courses, which are in every way suitable for graduate students. All further information may be obtained on application to the Hon. Secretary, Dr. H. Strauss, Kurfürstendamm 239, Berlin W., or Herr Melzer, Ziegelstrasse 10-11, Berlin.

The annual meeting of the After-Care Association for Poor Persons Discharged Recovered from Asylums for the Insane was held on Wednesday afternoon, the 16th inst., at the Royal College of Physicians. Sir R. Douglas Powell, President of the Royal College of Physicians, presided at the meeting, and there was a large attendance. During the past year 348 applications were dealt with. The receipts were £1,273, while the expenditure amounted to £858. The Bishop of Rochester moved the adoption of the report and balance-sheet, and said the association covered an area in philanthropy which was entirely its own, and had, therefore, a special claim upon the support of the community. Dr. G. H. Savage, honorary treasurer and one of the trustees, seconded the resolution. Sir William Collins, M.P., in cordially commending the work of the association to the support of the public, said we had too much neglected the importance of dealing with the transition stage of disease of the mind and complete sanity.

In the Dublin King's Bench Division on February 8, the jury, by direction of the Lord Chief Baron, found for the defendants in a case in which Mr. Myles Keogh sued the Governors of the Incorporated Dental Hospital of Ireland to recover £5,000 damages in respect of their refusal to admit him as a student of the hospital. In May 1908 the Governors passed the following resolution, on which the action was grounded : " Resolved, that Mr. Keogh cannot be accepted as a student at the hospital, the Committee having the right by their laws to refuse any student without assigning a cause." Plaintiff put on these words the construction that he was unfit and unworthy to attend the lectures, and was not a proper person to be admitted a student at the hospital. The defendants pleaded that the words did not bear any of the meanings placed on them by the plaintiff; that the publication was made *bona fide*, in the belief that the words were true, without malice, and on a privileged occasion. The Lord Chief Baron held that privilege had been proved. Neither was there any evidence that the publication was with malice.

Sir James Crichton-Browne, M.D., LL.D., F.R.S., will preside at the annual dinner of the London School of Clinical Medicine (Postgraduate), at Princes' Restaurant, on March 15.

Mr. Keetley, F.R.C.S., late senior surgeon of the West London Hospital, who died on December 4, aged sixty-one, left estate valued for probate at £2,267 10s. 4d. gross, of which £1,154 12s. 10d. is net personalty.

A public meeting was held at the Royal United Service Institution, Whitehall, on Monday, February 14, in connection with the annual general meeting of the Incorporated Institute of Hygiene, of Devonshire Street, Harley Street, W. Sir William Bennett, K.C.V.O., F.R.C.S. (the President of the Institute), took the chair, and the audience was largely representative of medicine, science, and education. The annual report indicated the efforts made to inculcate the principles of health and hygiene and to awaken interest in the subject. A large number of lectures have been delivered in London, the Midlands, and the North of England, and conferences have been held on subjects affecting the public health. A permanent museum of hygiene, the first in the provinces, has also been opened at Birmingham, similar to that in London, where students and others interested can have the character and constituents of foods and other articles in everyday consumption and use explained and demonstrated. The examinations in hygiene, held periodically in the leading towns of England, Scotland, and Ireland, were specially referred to as of great and increasing importance owing to the fact that the candidates were chiefly school-teachers, who were consequently in a position to impart much of their acquired knowledge to the young. The report further dealt with the necessity and urgency of extending the work of the Institute in regard to the promotion of hygiene and preservation of health.

At Manchester last week an action brought by a cotton weaver, against Dr. W. Price Grant, of Rochdale, to recover damages for alleged negligence in certifying her to be of unsound mind, and thus causing her detention in the County Lunatic Asylum at Lancaster for more than twelve months, came before Mr. Justice Walton. Mr. Taylor, for Dr. Grant, said he and Mr. Sutton, who appeared for the plaintiff, acting in the best interests of their clients and in the general interest, had agreed that there should be an end of the litigation. On behalf of Dr. Grant he had to say that they had come to the conclusion, after hearing the evidence, that the woman was not insane. He understood that Mr. Sutton acquitted the defendant of want of care in making his examination. It must be understood that the result of the litigation was to free Dr. Grant from any charge of having acted with negligence. Mr. Justice Walton said it was now admitted that all reflections on Dr. Grant were withdrawn, and, he thought, properly withdrawn. This case had brought home to his mind very vividly that the gravest responsibility rested upon constables, relieving officers, magistrates, and medical men, who put into force the machinery of the lunacy law. He was not reflecting upon anything which had been done in this case, but it seemed plain that a man or a woman, not a pauper, but perfectly well able to maintain himself or herself, might be taken to the workhouse, shut up in an imbecile ward, and then declared to be a lunatic and placed in a lunatic asylum without any public inquiry, any hearing before a magistrate, or any notice of what was going on. All that being possible, the gravest responsibility rested upon those who administered the law.

The King has been pleased to appoint Sir Thomas Myles, M.D., B.C., to be one of the honorary surgeons to his Majesty in Ireland in the room of Sir William Thomson, C.B., M.D., deceased.

His Majesty the King has accepted a copy of the inaugural address delivered by Dr. George Burford on "The Medicine of the Future: Coming Events that Cast their Shadows Before," at the opening of the winter session of the Honyman-Gillespie Lecture Courses at the London Homœopathic Hospital, Great Ormond Street, London, W.C.

Sir Walter Foster, M.D., has resigned his seat in Parliament in order that Colonel Seeley may have an opportunity of contesting it.

The Mary Kingsley medal of the Liverpool School of Tropical Medicine has been awarded to the following:—Mrs. Pinnock, in recognition of the services rendered to the cause of tropical medicine and sanitation by her brother, the late Sir Alfred Jones, founder and first chairman of the school; Mr. William Adamson and Professor William Carter, for assistance rendered in the foundation of the school; Prince Auguste d'Arenberg, president of the Suez Canal Company, for his campaign against malaria at Ismailia; Sir William Macgregor, Governor of Queensland, for his services to sanitation and tropical medicine while Governor of Lagos; Surgeon-General Walter Wyman, head of the Marine Hospital Service of the United States, for the organisation which he has given to the service under him and for the manner in which he has always supported scientific principles in public sanitation. Sir Alfred Keogh, recently Director-General of the Royal Army Medical Corps, for the organisation which he has given to the service under him and for the manner in which he has always supported scientific principles in public sanitation. The following have been awarded the medal for valuable contributions to the scientific and educational side of tropical medicine:—Professor R. Blanchard, Paris; Dr. Anton Breiul, recently director of the Research Laboratories of the School at Runcorn, and now director of the Tropical Diseases Institute in Queensland; Professor Angelo Celli, of Rome; Dr. C. W. Daniels, director of the London School of Tropical Medicine; Surgeon-Colonel King, Indian Medical Service; Professor Dr. Nocht, director of the Hamburg School of Tropical Medicine; Professor G. H. F. Nuttall, Quick Professor of Parasitology at Cambridge, and External Examiner in Tropical Medicine to the University of Liverpool; Major Leonard Rogers, Indian Medical Service; Professor J. L. Todd, formerly director of the Research Laboratories of the School at Runcorn, and now associate Professor of Parasitology at McGill University.

